# Ethical Governance of Artificial Intelligence in Nursing Management: Frameworks, Challenges, and Leadership Imperatives

**DOI:** 10.1155/jonm/4870265

**Published:** 2026-11-09

**Authors:** Serena K. Tobar

**Affiliations:** ^1^ Memorial Hermann Southwest Hospital 7600 Beechnut St Houston 77074 Texas USA

**Keywords:** algorithmic bias, artificial intelligence, digital health, ethical governance, healthcare administration, nurse leadership, nursing management, workforce equity

## Abstract

Artificial intelligence (AI) is increasingly embedded in healthcare organizations, influencing workforce management, operational decision‐making, quality monitoring, and resource allocation. While AI offers opportunities to improve efficiency and organizational performance, its implementation also introduces significant ethical challenges related to privacy, transparency, accountability, bias, and workforce equity. Nurse leaders occupy a pivotal position in shaping how AI is integrated into healthcare systems and ensuring that technological innovation aligns with professional nursing values. This editorial examines ethical AI governance as a leadership imperative in nursing management, drawing on the American Nurses Association Code of Ethics, World Health Organization guidance, leadership theory, sociotechnical systems theory, and equity‐centered scholarship. Key ethical concerns associated with AI‐enabled management are explored, including algorithmic bias, workforce surveillance, explainability, and accountability in decision‐making. The discussion highlights the responsibility of nurse managers to establish governance structures that protect human dignity, promote justice, and preserve professional judgment. To support this work, the Ethical AI Governance Framework for Nursing Management (EAG‐NM) is proposed as a conceptual model integrating ethical foundations, leadership imperatives, sociotechnical dynamics, equity safeguards, and organizational governance. The framework positions nurse leaders as ethical stewards of AI adoption and emphasizes workforce readiness, interdisciplinary oversight, and organizational accountability. As AI continues to transform healthcare operations, nursing leadership must move beyond technology implementation toward intentional governance that ensures AI is deployed in ways that are transparent, equitable, and consistent with the profession’s moral commitments.

## 1. Introduction

Artificial intelligence (AI) has moved from a conceptual possibility to an operational reality in healthcare organizations. Predictive analytics inform staffing decisions, natural language processing tools streamline documentation, and machine learning models support quality and safety monitoring. These technologies promise to reduce administrative burden, enhance efficiency, and improve organizational performance. Yet their integration also introduces ethical complexities that directly affect workforce equity, patient outcomes, and institutional trust.

Nursing leadership sits at the center of this transformation. Nurse managers are responsible not only for operationalizing AI tools but also for ensuring that their use aligns with the profession’s ethical commitments. The 2025 American Nurses Association (ANA) Code of Ethics underscores this responsibility by emphasizing the need for nurses to engage critically with emerging technologies, advocate for transparency, and address structural inequities that digital systems may amplify [1]. Provisions 7 through 9 explicitly call for ethical vigilance in adopting technologies that influence care delivery, workforce dynamics, and organizational decision‐making.

This article reframes the ethical use of AI as a leadership and governance imperative. Rather than focusing on research ethics or technical considerations, the discussion centers on the managerial responsibilities required to ensure that AI systems support equitable, person‐centered, and ethically grounded organizational practices. Drawing on the ANA Code of Ethics, World Health Organization (WHO) governance principles, leadership theory, sociotechnical systems theory, and equity‐centered scholarship, this article offers a comprehensive analysis of the ethical challenges and leadership strategies needed for responsible AI integration in nursing management.

## 2. AI in Nursing Management: Transforming Organizational Decision‐Making

AI technologies are increasingly integrated into the operational infrastructure of healthcare organizations. Machine learning models forecast staffing needs using historical patterns, patient acuity, and seasonal trends. Predictive analytics identify patients at risk of deterioration, enabling leaders to allocate resources proactively. Natural language processing tools extract insights from clinical documentation, supporting quality improvement and regulatory reporting [2, 3].

These tools offer significant benefits. They can reduce administrative workload, enhance the precision of managerial decisions, and support more equitable distribution of resources [2, 3]. For example, AI‐supported staffing systems can analyze real‐time patient acuity and workforce availability to recommend optimal staffing patterns, improving both efficiency and equity [4]. Decision‐support dashboards can synthesize large volumes of data to help leaders identify trends in safety events, patient satisfaction, or workforce turnover, strengthening organizational insight and responsiveness [3].

However, the effectiveness of these tools depends on the ethical integrity of the systems and the leadership structures that govern them. Without intentional oversight, AI can inadvertently perpetuate inequities, compromise privacy, or erode staff trust. Thus, integrating AI into nursing management requires not only technical competence but also ethical vigilance, organizational accountability, and a commitment to justice.

Recent empirical work reinforces these trends. Elkholy et al. [5] found that nurse managers often have limited AI readiness, with “more than two‐thirds (66.8%) having a low level of perception regarding overall AI,” despite recognizing its relevance to operational efficiency. Their study also demonstrated “a positive statistically significant correlation between nurse managers’ overall perception of AI and health logistics management,” underscoring the managerial importance of AI literacy for effective resource coordination and workflow oversight.

## 3. Theoretical Foundations for Ethical AI Governance in Nursing Management

### 3.1. Nursing Ethics as a Leadership Framework

The 2025 ANA Code of Ethics establishes the moral grounding for AI governance by emphasizing dignity, justice, and the protection of vulnerable populations. Provisions 7–9 call for ethical engagement with emerging technologies and position nurse leaders as stewards of transparency and equity. These ethical commitments translate directly into managerial practice through established leadership theories.

Ethical leadership provides a foundation for evaluating algorithmic decisions with fairness and moral courage, especially when communicating AI‐supported recommendations to staff [6]. Transformational leadership supports the cultural adaptation needed for AI integration by fostering trust, a shared vision, and psychological safety [7], all essential for navigating technological change. Authentic leadership reinforces relational transparency and integrity [8], enabling leaders to openly acknowledge uncertainty, discuss AI limitations, and model ethical decision‐making.

Together, nursing ethics and leadership theory form a coherent framework for guiding AI adoption in ways that preserve professional judgment, protect workforce dignity, and ensure alignment with nursing values. The ANA Ethics Advisory Board [9] underscores this responsibility, emphasizing that nurses must critically assess AI systems and advocate for their ethical use.

Although the ANA Code provides an American‐based foundation, ethical governance principles vary internationally. Many health systems share commitments to transparency, justice, and accountability, yet differ in regulatory structures, data governance requirements, and cultural expectations. Recognizing this diversity strengthens the global relevance of ethical AI leadership and highlights the need for context‐sensitive governance.

### 3.2. Organizational Ethics and Leadership Accountability

AI adoption reflects organizational priorities, power structures, and governance practices. Leadership theories, including ethical, transformational, and authentic leadership, offer practical guidance for how nurse managers can responsibly integrate AI into managerial workflows.

Ethical leadership emphasizes fairness, transparency, and moral courage, qualities essential for navigating algorithmic opacity and potential bias. Transformational leadership supports the cultural and behavioral change required for AI implementation by cultivating trust, psychological safety, and shared purpose. Authentic leadership reinforces integrity and relational transparency, enabling leaders to guide teams through uncertainty and maintain alignment with organizational values.

Collectively, these leadership approaches position nurse managers as ethical stewards who must balance innovation with accountability. Their role is to ensure that AI enhances organizational performance without compromising human dignity, equity, or the relational foundations of nursing practice.

### 3.3. AI‐Specific Leadership in Nursing Management

The integration of AI is redefining leadership practice by introducing new forms of decision authority, data‐driven reasoning, and human–machine collaboration. Traditional leadership theories remain relevant but must evolve toward digital and human–AI leadership, which emphasize technological fluency, ethical oversight, and relational adaptability. Recent work on nurse‐led AI governance shows that leaders need competencies in risk mitigation, interdisciplinary partnership, and digital decision rights to guide safe, scalable adoption [10]. In AI‐enabled environments, nurse leaders no longer serve solely as interpreters of human judgment; they also mediate between algorithmic logic and clinical expertise. Ethical leadership equips them to question algorithmic recommendations, demand transparency from vendors, and ensure that data‐driven outputs align with professional values. Transformational leadership helps cultivate trust and psychological safety when staff express uncertainty about AI decisions. Authentic leadership supports relational transparency by acknowledging both the promise and limitations of AI while modeling reflective skepticism rather than blind reliance.

Emerging scholarship on digital leadership and human–AI collaboration suggests that effective leaders balance technological competence with moral discernment. They translate complex algorithmic insights into accessible language, facilitate interdisciplinary dialog, and uphold accountability when automated decisions conflict with clinical intuition. This evolving leadership identity, the human–AI ethical leader, embodies nursing’s moral commitments while navigating the sociotechnical realities of digital transformation [11].

### 3.4. Sociotechnical Systems Theory: Understanding AI as Human–Technology Interaction

AI systems are not merely technical tools; they are components of sociotechnical systems where human, organizational, and technological elements interact. Sociotechnical theory emphasizes that technology both shapes and is shaped by workflows, culture, policies, and power dynamics [12].

This perspective is essential for nursing management because it highlights

• AI’s potential to redistribute power between leaders and staff.
• The risk of unintended consequences when algorithms interact with complex human systems.
• The need for continuous monitoring, adaptation, and human oversight.
• The importance of aligning AI with organizational values and workforce needs.

By applying sociotechnical theory, nurse leaders can anticipate how AI will affect communication patterns, decision‐making, and team dynamics. Jordan et al. [13] found that emergency nurses initially questioned AI triage alerts but later described them as a “safety net,” illustrating how AI reshapes communication, trust, and workflow dynamics in complex sociotechnical environments. This theoretical lens underscores the need for interdisciplinary governance, transparent communication, and ongoing evaluation.

### 3.5. Equity‐Centered and Critical Perspectives on AI

Scholars such as Shaw et al. [14, 15] and Skorburg et al. [16] highlight the ethical risks of algorithmic bias, data extraction, and the reduction of individuals to data points. These perspectives align with nursing’s longstanding commitment to social justice and provide a critical lens for evaluating AI’s impact on marginalized populations.

For nurse leaders, equity‐centered frameworks require:

• Examining how AI may reinforce structural inequities.
• Ensuring diverse data representation.
• Advocating for inclusive design and evaluation processes.
• Protecting vulnerable groups from harm or exploitation.

These perspectives deepen the ethical analysis by situating AI within broader social and political contexts, reinforcing the need for justice‐oriented leadership.

Nurse leaders can anticipate how AI will affect communication patterns, decision‐making processes, and team dynamics because they oversee the daily coordination of clinical workflows and understand how information moves through the organization. Their proximity to frontline staff gives them insight into how changes in documentation systems, decision‐support tools, or automated alerts reshape interpersonal communication and workload distribution. As managers responsible for staffing, resource allocation, and performance oversight, nurse leaders are uniquely positioned to identify when AI‐enabled processes disrupt collaboration, create bottlenecks, or shift decision authority in ways that affect team functioning.

## 4. Ethical Challenges in AI‐Enabled Nursing Management

### 4.1. Data Privacy and Workforce Confidentiality

AI systems rely on vast amounts of data, including sensitive workforce and patient information. Predictive analytics may track staff performance, identify patterns in documentation, or monitor workflow efficiency, raising important questions about how these data are collected, interpreted, and used within organizations [14]. While these tools can support organizational improvement, they also raise ethical concerns about surveillance, autonomy, and confidentiality.

Scholars caution that data‐intensive AI systems risk reducing individuals to mere data points and may compromise personhood and agency without robust safeguards [3, 16]. These concerns align with global AI ethics frameworks, which consistently identify privacy and surveillance as critical risks that require strong governance [17].

### 4.2. Algorithmic Bias and Workforce Equity

Algorithmic bias poses one of the most significant ethical risks in AI‐enabled management. AI models trained on incomplete or biased datasets can produce inequitable staffing patterns, unfair workload distribution, or biased performance evaluations. Shaw et al. [14] highlight the global implications of algorithmic bias, noting that inequitable outcomes can disproportionately affect marginalized populations. Ruksakulpiwat et al. [4] emphasize the need for inclusive data representation and equitable model design.

Bodur et al. [18] found that nurses feared AI could erode relational care and dehumanize practice, reinforcing the need for justice‐oriented and relationally grounded governance. For nurse leaders, addressing algorithmic bias requires ongoing auditing, interdisciplinary collaboration, and a commitment to equity. Leaders must ensure that AI systems do not reinforce existing disparities or create new forms of inequity within the workforce.

### 4.3. Transparency and Explainability in Managerial Decision‐Support

Opaque AI systems undermine trust and hinder ethical decision‐making. When algorithms influence staffing, scheduling, or quality metrics, leaders must be able to explain how decisions are generated, what data informed the model, and what limitations apply. Transparency and explainability are essential to maintaining workforce confidence and ensuring that AI‐supported decisions align with organizational values [14, 19]. Wieben et al. [20] found that nurses were reluctant to rely on AI recommendations unless they understood how the system generated them, reinforcing the importance of explainability for preserving autonomy and ethical decision‐making.

Healthcare leaders consistently report that insufficient insight into algorithmic processes creates uncertainty and erodes trust, underscoring the need for clear communication and robust governance structures [3]. These concerns align with global AI ethics frameworks, which identify transparency as a foundational requirement for responsible AI adoption [17].

### 4.4. Accountability in AI‐Driven Decisions

As AI becomes more integrated into managerial workflows, questions of accountability become increasingly complex. Shaw et al. [14] argue for transparent governance structures that clarify responsibility when AI‐supported decisions lead to errors or inequities. Nurse leaders must ensure that human oversight remains central; that decision pathways are documented; and that accountability is shared across leadership, vendors, and technical teams.

While technical challenges such as data quality, model accuracy, and system integration shape the performance of AI tools, the ethical responsibilities associated with AI use fall primarily to the human agents who develop, implement, and oversee these systems. Technical teams are responsible for ensuring model validity, documenting limitations, and maintaining transparency about data sources and algorithmic behavior. Nurse leaders, in contrast, hold ethical responsibilities related to fairness, accountability, workforce protection, and alignment with professional values. Distinguishing these roles clarifies that ethical governance is not a technical function but a leadership obligation that requires critical evaluation, structured oversight, and ongoing dialog with technical experts.

A recent scoping review by Katebi et al. [21] underscores these ethical challenges, noting that AI in nursing management is increasingly used for “data‐driven decision‐making, workflow automation, staffing optimization, and patient monitoring,” yet its implementation is constrained by data privacy concerns, algorithmic bias, staff resistance, and interoperability limitations. These findings align with global concerns about transparency, fairness, and the need for robust governance structures to support responsible AI adoption.

## 5. Organizational Oversight and Governance Structures

### 5.1. AI Oversight Committees

Healthcare organizations should establish interdisciplinary AI oversight committees to evaluate AI tools, monitor performance, and ensure alignment with ethical principles. These committees can review vendor algorithms, conduct bias audits, oversee implementation, and develop organizational AI policies. NICU nurses expressed concerns about reliability, workflow disruption, and insufficient governance structures [22], highlighting the need for robust oversight mechanisms. The committee’s work ensures that AI adoption is intentional, equitable, and aligned with organizational values.

### 5.2. Ethical Procurement and Vendor Evaluation

Ethical AI governance begins with procurement. Nurse leaders must advocate for procurement processes that prioritize transparency, equity, and safety. This includes requiring vendors to disclose training data sources, model limitations, bias mitigation strategies, and data governance practices. Ethical procurement ensures that AI systems are evaluated not only for technical performance but also for their ethical implications.

### 5.3. Policy Development and Ethical Safeguards

Organizational policies should address data privacy, workforce protections, algorithmic auditing, documentation of AI‐supported decisions, and procedures for reporting concerns. These policies provide a framework for ethical AI use and ensure that staff understand their rights and responsibilities.

## 6. Workforce Readiness and Ethical AI Literacy

### 6.1. Education and Training for Ethical AI Use

Nurse leaders play a critical role in preparing the workforce for AI‐enabled environments. Education must extend beyond technical instruction to include ethical considerations such as algorithmic bias, data privacy, and the limitations of predictive analytics. Microcredentialing programs from the Healthcare Information and Management Systems Society (HIMSS) [23] offer accessible pathways to build AI literacy and support lifelong learning.

Developing ethical AI literacy is essential to prepare the workforce to engage with increasingly complex digital tools. The ANA Ethics Advisory Board [9] emphasizes that nurses must understand both the capabilities and limitations of AI systems to evaluate their ethical implications and advocate for responsible use. Organizational support and peer influence significantly predicted positive attitudes toward AI adoption [24]. This underscores the need for comprehensive education that integrates technical knowledge with ethical reasoning and professional accountability supported by nursing leadership.

Competency development frameworks can further support nurse managers in preparing for AI‐enabled environments. The American Organization for Nursing Leadership [25] competency domains of communication, knowledge management, relationship building, and systems thinking closely align with the skills required for ethical AI governance. Integrating AI‐specific competencies into these frameworks can help leaders assess readiness, identify learning needs, and structure professional development initiatives that support responsible AI adoption.

### 6.2. Psychological Safety and Change Management

Integrating AI ethically requires a psychologically safe environment where staff can raise concerns about fairness, accuracy, and potential unintended outcomes. Leadership plays a critical role in fostering open communication, addressing apprehensions about job displacement, and clarifying that AI is intended to enhance, not replace, professional judgment. Transparent, well‐structured change management processes are essential to support this transition.

### 6.3. Implications for Nursing Leadership

Nurse leaders are uniquely positioned to guide ethical AI adoption because they serve as the primary link among organizational strategy, clinical operations, and workforce experience. Their responsibilities include interpreting institutional priorities, translating them into practice, and ensuring that technological changes support safe, equitable care delivery. Unlike technical teams or external vendors, nurse leaders understand the relational, ethical, and contextual dimensions of nursing practice. This enables them to assess whether AI systems enhance professional judgment, support equitable workload distribution, and align with the values that guide nursing care.

The ANA Ethics Advisory Board [9] argues that the ethical use of AI requires intentional leadership and a commitment to ensuring that technological innovation aligns with nursing’s core values. This reinforces the need for governance structures that safeguard equity, transparency, and accountability as AI becomes increasingly embedded in organizational decision‐making.

Qualitative findings further emphasize the centrality of nurse managers in AI governance. Göktepe and Sarıköse [26] found that “nurse managers play a critical role in overseeing nursing workflows, ensuring patient safety, and leading organizational change,” and that AI integration is shaped by managerial strategies that address staff concerns, workflow redesign, and ethical oversight. Their participants also described infrastructure deficiencies, data security concerns, and cultural resistance as barriers to implementation, reinforcing the need for leadership‐driven governance structures that anticipate organizational and workforce challenges.

Taken together, these perspectives reveal that ethical AI governance requires more than applying existing leadership theories to new technologies. It represents a shift in leadership practice that integrates ethical reasoning, digital literacy, sociotechnical awareness, and equity‐centered vigilance. To clarify how these elements interact, this editorial proposes the Ethical AI Governance Framework for Nursing Management (EAG‐NM), a model that synthesizes ethical foundations, leadership imperatives, sociotechnical dynamics, equity safeguards, and organizational governance into a coherent structure for guiding AI adoption.

## 7. EAG‐NM

### 7.1. A Unifying Conceptual Model for Ethical and Operational AI Leadership

The EAG‐NM positions nurse leaders as the central ethical stewards of AI adoption by integrating five mutually reinforcing domains. Together, these domains explain how and why nurse managers are uniquely equipped to govern AI ethically and operationally.

#### 7.1.1. Ethical Foundations (Professional and Global Ethics)

Core question: What moral commitments guide AI use in nursing management?

This domain anchors governance in

• The ANA Code of Ethics (2025): dignity, justice, transparency, and protection of vulnerable populations.
• Global ethics variation: WHO guidance, international differences in governance, and cultural expectations.
• Nursing ethics as leadership ethics: ethical vigilance, preservation of professional judgment, and advocacy for equity.

This domain establishes the why of ethical AI governance.

#### 7.1.2. Leadership Imperatives (Ethical, Transformational, and Authentic)

Core question: What leadership behaviors operationalize ethical AI governance?

This domain explains how nurse leaders enact ethical principles through:

• Ethical leadership: fairness, transparency, and moral courage.
• Transformational leadership: trust‐building, shared vision, and psychological safety.
• Authentic leadership: relational transparency, integrity, and acknowledgment of uncertainty.

This domain shows why nurse leaders, rather than vendors or IT departments, are uniquely positioned to guide ethical adoption: they are the only leaders who understand clinical realities, workforce dynamics, and organizational ethics.

#### 7.1.3. Sociotechnical Integration (Human–Technology Interaction)

Core question: How does AI reshape workflows, communication, and decision‐making?

This domain highlights:

• AI as part of a complex sociotechnical system.
• Redistribution of decision authority.
• Workflow disruptions and unintended consequences.
• The need for continuous monitoring and human oversight.

Evidence from Jordan et al. [13], Wieben et al. [20], and Göktepe and Sarıköse [26] demonstrates how AI alters communication patterns, trust, and team dynamics.

#### 7.1.4. Equity and Justice Safeguards (Bias, Representation, and Vulnerability)

Core question: How do leaders ensure AI does not reproduce or amplify inequities?

This domain integrates:

• Algorithmic bias risks (Shaw et al. and Skorburg et al.).
• Workforce equity concerns (Bodur et al. and Ruksakulpiwat et al.).
• Protection of marginalized groups.
• Inclusive data governance and bias auditing.

This domain underscores that ethical AI governance is not only technical but is also a fundamentally justice work.

#### 7.1.5. Organizational Governance and Workforce Readiness

Core question: What structures and competencies make ethical AI adoption possible?

This domain includes:

• AI oversight committees.
• Ethical procurement.
• Policy development.
• Competency frameworks (AONL).
• AI literacy, training, and psychological safety.
• Change management strategies.

Evidence from Elkholy et al. [5], Amin et al. [24], Ayed et al. [22], and Katebi et al. [21] shows that readiness gaps are widespread and leadership‐driven solutions are essential.

Figure 1 presents the EAG‐NM, showing the relationships among ethical foundations, leadership behaviors, sociotechnical dynamics, equity safeguards, and organizational governance.

**FIGURE 1:**
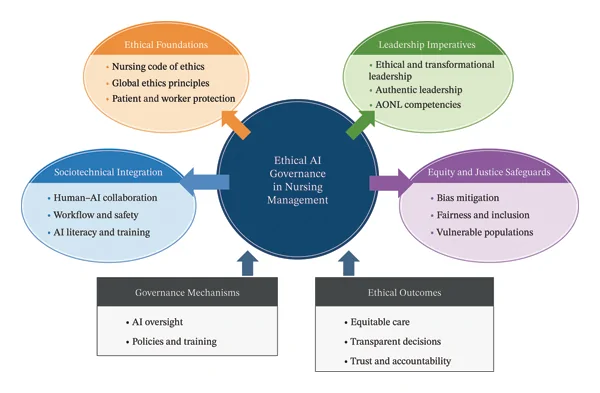
Ethical AI Governance Framework for Nursing Management (EAG‐NM): a conceptual model for nurse leader stewardship of artificial intelligence. This conceptual framework depicts the relationships among ethical foundations, leadership imperatives, sociotechnical integration, equity and justice safeguards, governance mechanisms, and ethical outcomes that collectively support ethical AI governance in nursing management. The model emphasizes the role of nurse leaders in ensuring that AI‐enabled decision‐making aligns with professional values, organizational accountability, workforce well‐being, and equitable patient care.

## 8. Conclusion

As AI becomes increasingly embedded in nursing management, future research should examine how leaders evaluate algorithmic outputs, how AI influences managerial decision‐making over time, and which governance structures most effectively mitigate bias and protect workforce autonomy. Additional studies are needed to explore cross‐cultural differences in AI governance, the impact of AI on leadership competencies, and the development of validated tools for assessing ethical readiness in AI‐enabled organizations. These research priorities will help build an evidence base that supports safe, equitable, and ethically grounded AI integration in nursing management.

## Funding

This work did not receive any external funding.

## Ethics Statement

This manuscript did not involve human subjects research, data collection, or participant interaction and therefore did not require institutional review board (IRB) approval. However, the ethical issues addressed in this article are grounded in established professional frameworks, including the ANA Code of Ethics for Nurses [1, 27] and the World Health Organization’s Ethics and Governance of Artificial Intelligence for Health (2021). These documents provide the ethical foundation for evaluating the responsibilities of nurse leaders in the governance of AI‐enabled systems.

The analysis also draws on contemporary scholarship examining algorithmic bias, data privacy, transparency, and the ethical use of digital technologies in healthcare. In keeping with the ethical commitments of the nursing profession, this manuscript emphasizes the importance of protecting patient and workforce rights, promoting equity, and ensuring that AI systems are implemented in ways that uphold human dignity and organizational integrity. No proprietary, confidential, or identifiable information was used in the development of this manuscript.

## Conflicts of Interest

The author declares no conflicts of interest.

## Data Availability

No datasets were generated or analyzed for this conceptual manuscript, and therefore no data are available.
